# Thermal Vapor Deposition of a Hydrophobic and Gas-Permeable Membrane on Zirconium Phosphate Cation Exchanger: An Oral Sorbent for the Urea Removal of Kidney Failure

**DOI:** 10.1021/acs.langmuir.4c01877

**Published:** 2024-07-23

**Authors:** Yihan Song, Sang-Ho Ye, Stephen R. Ash, Lei Li

**Affiliations:** † Department of Chemical and Petroleum Engineering, University of Pittsburgh, Pittsburgh, Pennsylvania 15260, United States; ‡ McGowan Institute for Regenerative Medicine, Pittsburgh, Pennsylvania 15210, United States; ⊥ Department of Surgery, University of Pittsburgh, Pittsburgh, Pennsylvania 15260, United States; § Nephrology Department, Indiana University Health Arnett Hospital, Lafayette, Indiana 47905, United States; ∥ CEO, HemoCleanse Technologies, LLC, Lafayette, Indiana 47904, United States

## Abstract

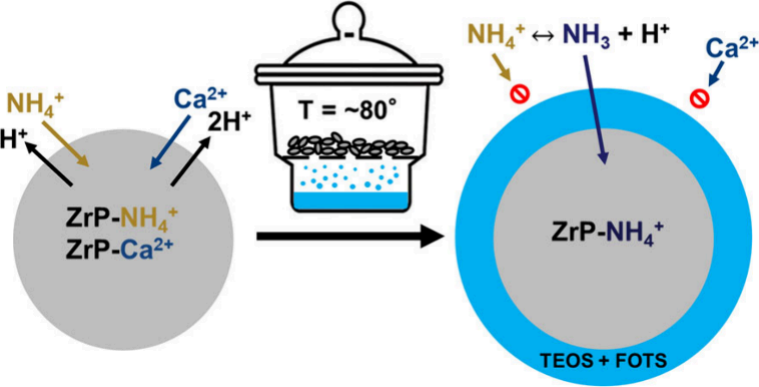

An oral sorbent with high capacity for NH_4_^+^ is desirable in lowering the blood urea level and mitigating the dialysis burden for end-stage kidney disease (ESKD) patients. Zirconium phosphate (ZrP) is an amorphous cation ion exchanger with high NH_4_^+^ binding capacity as a sorbent material, but its selectivity to remove NH_4_^+^ is limited in the presence of other competing ions in water solution. We previously have developed a gas-permeable and hydrophobic perfluorocarbon coating on ZrP, which improves ZrP’s NH_4_^+^ selectivity. However, the coating preparation procedure, a wet chemistry approach, is complicated and time-consuming, and more importantly, the large amount of usage of acetone poses a concern for the application of ZrP as an oral sorbent. In this study, we developed a solventless coating protocol that effectively coats ZrP with tetraethyl orthosilicate (TEOS) and 1*H*,1*H*,2*H*,2*H*-perfluorooctyltriethoxysilane (FOTS) via thermal vapor deposition (TVD) in a simplified manner. X-ray photoelectron spectroscopy (XPS) and contact angle measurements verify the two coatings are successfully deposited on the ZrP surface, and the coating condition was optimized based on an *in vitro* static binding study. The dynamic binding study of competing ions on Na-loaded ZrP with TVD coatings yields a maximum NH_4_^+^ removal (∼3.2 mequiv/g), which can be improved to ∼4.7 mequiv/g if H-loaded ZrP under the same coating condition is used in basic stock solutions. More importantly, both materials barely remove Ca^2+^ and show excellent acid resistance. The significant improvement in the NH_4_^+^ binding capacity and selectivity reported here establishes a highly promising surface modification approach to optimize oral sorbents for ESKD patients.

## 1 Introduction

Chronic kidney disease (CKD) is a rapidly growing global public health burden, affecting more than 10% of the worldwide population.^1,2^ Approximately 786,000 people in the United States are currently living with end-stage kidney disease (ESKD), which is defined as the final stage of CKD treated with dialysis or a kidney transplant.^3^ Kidney transplantation is considered as the optimal therapeutic approach for ESKD, but the supply of available kidneys cannot satisfy the current demand, along with the downsides of high graft failure rate and the ineligibility of many patients for this treatment.^4^ Hence, the majority of patients with ESKD rely on hemodialysis or peritoneal dialysis as a substitute for kidney function. However, patients undergoing hemodialysis are constrained by the need to visit the clinic three times a week, enduring up to 4 h per session, which restricts their freedom and autonomy.^5^ Unlike a healthy kidney that consistently maintains homeostasis, the intermittent nature of hemodialysis treatment results in the buildup of uremic waste solute, water, and electrolytes such as Na^+^, K^+^, and PO_4_^3–^, which are associated with conditions such as hypertension and cardiovascular disease.^4,5^ Peritoneal dialysis offers more continuous in-home treatment.^6,7^ However, its overall toxin removal efficiency is lower than hemodialysis, and the rapid functional decline of the peritoneal membrane in high-concentration glucose limits the technique’s lifetime.^6,7^ Thus, patients typically transition to hemodialysis as their treatment option after an average of 3.7 years.^8^ The conventional dialysis treatment results in considerable treatment burden, dietary restrictions, and poor clinical outcomes.^4,5,7,9,10^ Therefore, an improved ESKD treatment approach could benefit both the patient and clinician.

A wearable or highly portable artificial kidney (WAK or PAK) with light weight and high toxin clearance away from the clinic could improve patient life quality by increasing their mobility, freedom, and ability to engage in social and economic life.^4,11^ The “REDY sorbent system” was a PAK used in home dialysis from 1973 to 1994.^12^ Such devices incorporate zirconium phosphate (ZrP, a nonselective cation exchanger) sorbent into a dialysis circuit to continuously regenerate dialysate through the adsorption of ammonium ions resulting from enzymatic breakdown of urea.^4,12^ However, ZrP’s nonselectivity in the presence of other ions, including calcium, magnesium, and potassium, limits its binding capacity to 0.96 mequiv of NH_4_^+^/g of ZrP and lowers the level of these ions, which need to be infused back into the dialysate.^4^ The adsorbed ions are exchanged for hydrogen and sodium ions, and sodium release is a concern, as higher sodium concentration in the dialysate is associated with hypertension.^13^ Minimization of Ca^2+^ removal and Na^+^ release in the dialysate has complicated the WAK or PAK and increased its size and weight.^13,14^

An oral sorbent could effectively bind and remove small and charged uremic toxins in the gut (SCUT: K^+^, H^+^, phosphate, NH_4_^+^ from urea), and thus dialysis therapy for ESKD could be directed principally at removal of organic and protein-bound toxins.^15^ A charcoal column is all that would be needed to accomplish effective regeneration of the dialysate, thus leading to the greatly simplified construction of a WAK or PAK.^15^ For decades oral sorbents such as anion exchangers have been used to decrease serum phosphate in patients with CKD and ESKD.^16^ Zirconium oxide loaded with hydroxide (ZO-OH) is well tolerated in animal studies and effective in binding phosphate in the gut.^15^ Zirconium cyclosilicate (ZS), a monovalent-selective cation exchanger, is highly effective for removal of potassium.^17,18^ However, there is no oral sorbent for effectively removing NH_4_^+^ or urea from the gut. About 25% of the urea generated by the liver in normal patients diffuses into the small bowel lumen daily. This urea is catalyzed to ammonium ion (NH_4_^+^) and bicarbonate by urease, and the products return to the liver, which resynthesizes the urea. In patients with CKD and elevated blood urea nitrogen (BUN) levels, the transfer of urea to the gut increases in proportion to the BUN. Thus, an effective oral sorbent for binding NH_4_^+^ in the gut should lower the BUN level in CKD patients. The above-mentioned ZrP is an excellent NH_4_^+^ binder, but the presence of competing ions limits binding capacity.^4^ Several urea removal strategies, including enzymatic hydrolysis, electrochemical decomposition, and urea sorbents, have been studied for WAK, but these approaches have toxic side effects or limited removal capacity.^4^

Our previous works demonstrated that a nonselective cation exchanger, ZrP, could be coated with a gas-permeable and hydrophobic membrane to improve its binding capacity for NH_4_^+^ and selectivity in the presence of other ions.^5,12^ The hydrophobic coating acts as a barrier to ions in water solution interacting with the cation exchanger. NH_4_^+^ is in equilibrium with NH_3_ in any solution. The coating’s gas permeability allows gaseous NH_3_ to transfer to the H-loaded exchanger. NH_3_ could pass through the membrane and bind with H^+^ to form NH_4_^+^, and thus it could be trapped within the capsule. However, the procedure for creating these membranes within a liquid environment is complex and consumes large amounts of acetone, which is a concern when it comes to oral sorbents.

Vapor-phase deposition is an alternative to this wet chemistry coating method, in which silanes can be coated on a solid surface from precursors vaporized under an elevated temperature or reduced pressure. Previous works^19−23^ have demonstrated that a monolayer or multilayers could form in the vapor phase depending on the reaction conditions, the chemistry of the silane molecule, and the surface structure. In addition, surface silanization within the vapor phase has been found promising in many applications, such as superhydrophobicity,^24,25^ anticorrosion,^26,27^ antistiction,^28,29^ blood-contacting medical devices,^30,31^ and biosensors.^32−34^ In this work, we hypothesized that the hydrophobic and gas-permeable membrane can be grafted onto ZrP in the vapor phase, which has been achieved by a simple solventless thermal vapor deposition (TVD) process. *In vitro* competing ion tests indicate that coated ZrP has significantly improved NH_4_^+^ binding capacity and selectivity, suggesting that a solventless procedure, such as vapor-phase deposition, is more promising for developing oral sorbents for ESKD patients.

## 2 Experimental Section

### 2.1 Materials

Amorphous ZrP loaded with sodium or hydrogen in granular form was provided by HemoCleanse Technologies LLC (Lafayette, IN, USA). Tetraethyl orthosilicate (TEOS)-coated ZrP was prepared following a wet chemistry protocol reported previously.^5,12^ TEOS, 1*H*,1*H*,2*H*,2*H*-perfluorooctyltriethoxysilane (FOTS), HEPES sodium salt, acetone, calcium chloride, and ammonium chloride were purchased from Sigma-Aldrich (St. Louis, MO, USA). Hydrogen chloride was obtained from Fisher Scientific (Waltham, MA, USA). Colorimetric testing kits for measurement of total nitrogen and calcium were purchased from Teco Diagnostics (Anaheim, CA, USA). Cuvettes of 4.5 and 3 mL were purchased from Fisher Scientific (Waltham, MA, USA) and Cole-Parmer (Vernon Hills, IL, USA), respectively.

### 2.2 TVD Coating Preparation

**Figure 1:**
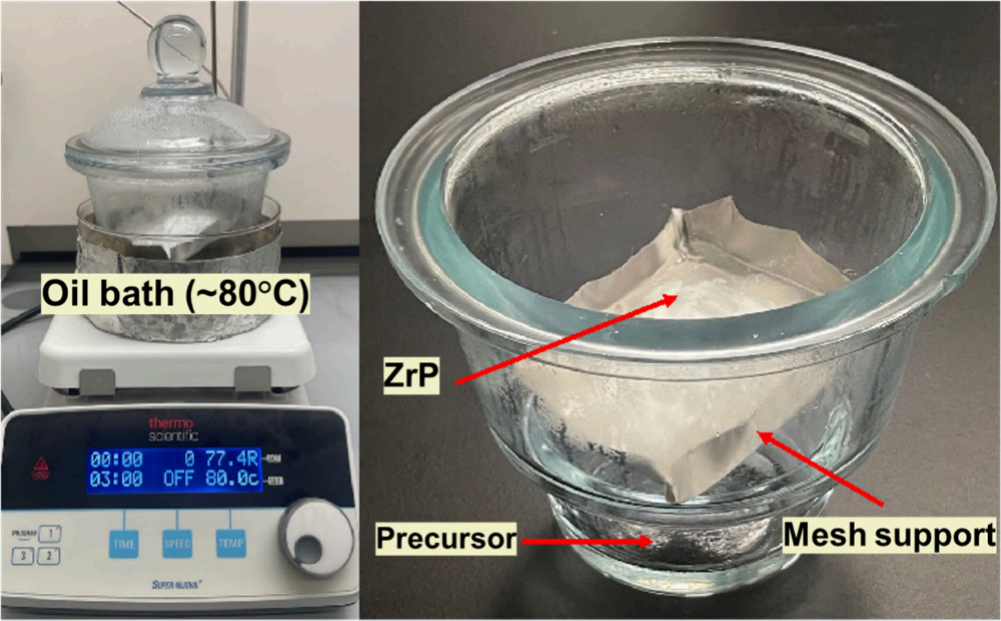
TVD setup showing the treatment of ZrP with TEOS or FOTS.

A simple TVD process was utilized to expose ZrP particles to the vapor of a coating precursor at elevated temperature (∼80 °C) for different durations. Using the TVD setup shown in Figure 1, 1 g of ZrP particles was placed on porous stainless-steel mesh in a closed chamber with an open vessel holding ∼1 mL of FOTS or ∼5 mL of TEOS for a duration ranging from 1 to 48 h at 80 °C. Coated ZrP materials are named as coating precursor (T/F) followed by TVD duration (1–48 h). For example, T24hF12h refers to ZrP coated by TEOS for 24 h and then by FOTS for 12 h, while TF12h indicates the ZrP material coated by solution-based TEOS and then vapor-phase FOTS for 12 h.

### 2.3 Acid Exposure Study

A HCl solution (pH = 2) was prepared, and 100 mg coated ZrP was added into a 20 mL solution in 50 mL centrifuge tubes.^12^ Capped tubes were placed upright for 3 h. The HCl solution was carefully removed from the tubes, and then, the coated ZrP was washed with DI water three times. The washed samples were air-dried in ambient conditions.

### 2.4 Characterization

#### XPS

The atomic surface composition of uncoated and coated ZrP materials was determined using XPS at the University of Washington’s Molecular Analysis Facility. Each sample was pressed flat onto a piece of double-sided Scotch tape that was adhered to a clean silicon wafer. Three spots on each sample were chosen for analysis. XPS spectra were taken on a Surface Science Instruments S-Probe spectrometer. This instrument has a monochromatized Al X-ray source and a low-energy electron flood gun for charge neutralization. X-ray spot size for these acquisitions was 800 × 800 μm or 200 × 200 μm. All samples were run as insulators. Pressure in the analytical chamber during spectral acquisition was about 5 × 10^–9^ Torr. Pass energy for survey spectra was 150 eV. A detailed scan was run for Zr, F, P, Si, and Na to improve quantification. Data analysis was carried out using the Service Physics Hawk Analysis 7 program (Service Physics, Bend, OR, USA).

#### SEM

SEM images of uncoated and coated ZrP materials were taken using a Zeiss SIGMA VP scanning electron microscope (Nanoscale Fabrication & Characterization Facility at the University of Pittsburgh) with the electron source of a Schottky thermal field emitter under high vacuum. The accelerating voltage utilized was 3 kV.

#### Contact Angle

The static and dynamic water contact angles of ZrP materials were measured using a VCA Optima XE (AST Production Inc., Billerica, MA, USA) system at room temperature and 48% humidity. Samples of ∼50 mg of each material were pressed into pellets using a pellet die. Static water contact angle was measured by the VCA software after placing a 1 or 2 μL droplet on the sample surface. The advancing water contact angle (WCA_a_) was measured by recording the continuous addition of water to a sessile drop (2 μL) for 5–8 s. Receding water contact angle (WCA_r_) measurements were taken by withdrawing water from the droplet for 8 s. The moment when the contact angle is maximum (minimum) is taken as the advancing (receding) contact angle: WCA_a_ is the moment right before the droplet width increases, and WCA_r_ is the moment right before the droplet width decreases.

#### Binding Study

*In vitro* competing ion studies were carried out to determine NH_4_^+^ binding capacity and selectivity of uncoated and coated ZrP materials in the presence of a competing ion.^12^ A testing solution containing 15 mM NH_4_^+^, 15 mM Ca^2+^, and 20 mM sodium based HEPES was prepared for this study. A 5 mL amount of testing solution and 50 mg of each ZrP material were placed into a 24 mL test tube, which was capped and placed on a shaker plate at 270 rpm. Each test tube was tested for the concentrations of NH_4_^+^ and Ca^2+^ via colorimetric analysis over a period of 24 h.

To mimic small intestine physiological conditions for ESKD patients, *in vitro* binding studies with continuously replacing the stock solution were conducted.^5^ A 5 mL amount of solution comprised 14 mM NH_4_^+^ and 12 mM Ca^2+^, and 50 mg of each ZrP material was placed into a 24 mL test tube, which was capped and placed on a shaker plate at 270 rpm. The solution in each test tube was replaced every 20 min for 5 h, and the three 20 min samples from each hour were combined to determine the concentrations of NH_4_^+^ and Ca^2+^ via colorimetric analysis.

Single ion binding studies were performed to determine the NH_4_^+^ equilibrium adsorption curves for uncoated and coated ZrP materials. Stock solutions were prepared to cover a range of concentrations: 15, 25, 35, 45, and 75 mM NH_4_^+^. HEPES (20 mM) was added to each solution to buffer the pH (∼8.3) during the binding studies. A 5 mL amount of solution and 50 mg of each ZrP material were placed into a 24 mL test tube, which was capped and placed on a shaker plate at 270 rpm. After 24 h, test tubes were removed from the shaker plate, and the NH_4_^+^ concentration of each tube was measured via colorimetric analysis.

#### Colorimetric Analysis

NH_4_^+^/NH_3_ and Ca^2+^ colorimetric testing kits obtained from Teco Diagnostics were used to quantify NH_4_^+^ and Ca^2+^ concentrations in stock solutions.^12^ Color development assays were prepared by following the instructions provided with each kit. NH_4_^+^ and Ca^2+^ concentrations were quantified using a Genesys S10 UV–vis monochromator in Dr. Haitao Liu’s Laboratory at the University of Pittsburgh. The wavelength of the monochromator was set to 570 nm for Ca^2+^ and 630 nm for NH_4_^+^ measurements. NH_4_^+^ and Ca^2+^ by sorbent materials was calculated from the depleted amount of NH_4_^+^ or Ca^2+^ in stock solutions.

## 3 Results and Discussion

### 3.1 Characteristics of Coated Na-Loaded ZrP

**Figure 2:**
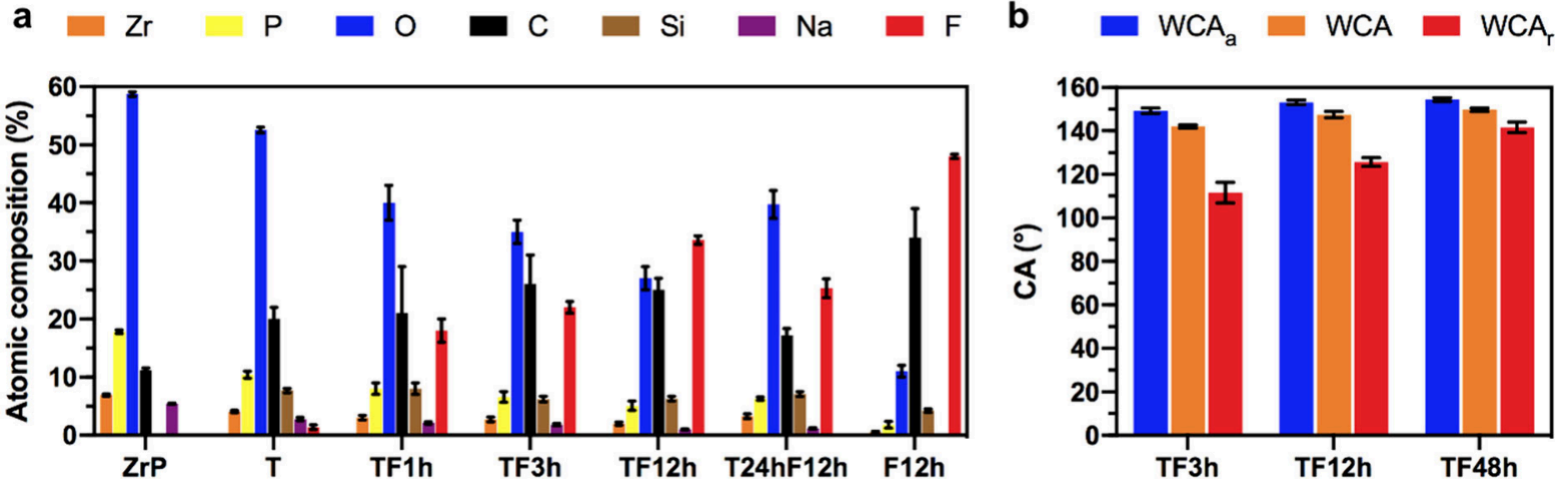
Atomic surface composition (a) and water contact angle (b) of ZrP with different coatings. “ZrP” is an uncoated material as the control. “T” refers to ZrP coated with TEOS in liquid phase, while “TF1h”, “TF3h”, and “TF12h” are ZrPs that were coated with TEOS in liquid phase followed by FOTS in vapor phase for 1, 3, and 12 h, respectively. “T24hF12h” was ZrP coated by TEOS vapor in 24 h exposure followed by FOTS vapor in 12 h exposure. “F12h” represents that ZrP was coated with FOTS only in vapor phase for 12 h.

XPS surface analysis results shown in Figure 2a include the atomic composition of uncoated and coated ZrP with different TVD conditions. The emergence of Si and F for coated materials with decreased Zr and P surface concentrations indicates TEOS and FOTS were successfully deposited on the ZrP surface. XPS has a takeoff angle of 90° and quantifies atoms to a depth of ∼10 nm beneath the surface. Thus, the coating thickness of TEOS plus FOTS is less than 10 nm, as indicated by the detection of some Zr and P. With increasing the TVD duration from 1 h to 12 h for FOTS coating, F composition increases from ∼18% to ∼34%. This is because longer TVD time allows more FOTS molecules to bombard and bond to the surface and thus guarantees more complete coating coverage to the ZrP surface. The dependence of surface coverage on reaction duration agrees with previous studies.^22,35^ T24hF12h has the TEOS coating step performed in TVD as well, which shows a lower composition of C (∼17%) and F (∼25%) but higher composition of O (40%) by comparison to TF12h (C ≈ 25%, F ≈ 34%, and O ≈ 27%,). This could be attributed to adsorption of TVD reaction byproducts (e.g., H_2_O and C_2_H_5_OH) onto the ZrP surface because our TVD is not followed by a washing step. F12h is a ZrP material coated with FOTS only, which has an atomic surface composition nearly equivalent to the theoretical composition of FOTS (F = 52%, C = 32%, Si = 4%, O = 12%). The Zr and P compositions on this material are negligible (<2%), indicating FOTS coating alone on ZrP gives rise to a thickness of at least 10 nm.

The results of the WCA measurement on coated ZrP materials are shown in Figure 2b. TF3h, TF12h, and TF48h have a WCA of ∼142°, ∼147°, and 149°, respectively, indicating the successful deposition of FOTS on the ZrP surface, and all coated materials are very hydrophobic. The advancing (WCA_a_) and receding (WCA_r_) contact angles of these materials were also measured. The WCA_a_ is ∼151° for all 3 materials, but WCA_r_ becomes higher for ZrP coated with longer TVD duration, leading to smaller difference in contact angle hysteresis (WCA_a_ – WCA_r_: ∼38° for TF3h and ∼13° for TF48h). This can be explained by the fact that a shorter TVD duration results in incomplete surface coverage of the FOTS coating, and those defects play an important role in WCA_r_ measurements.

**Figure 3:**
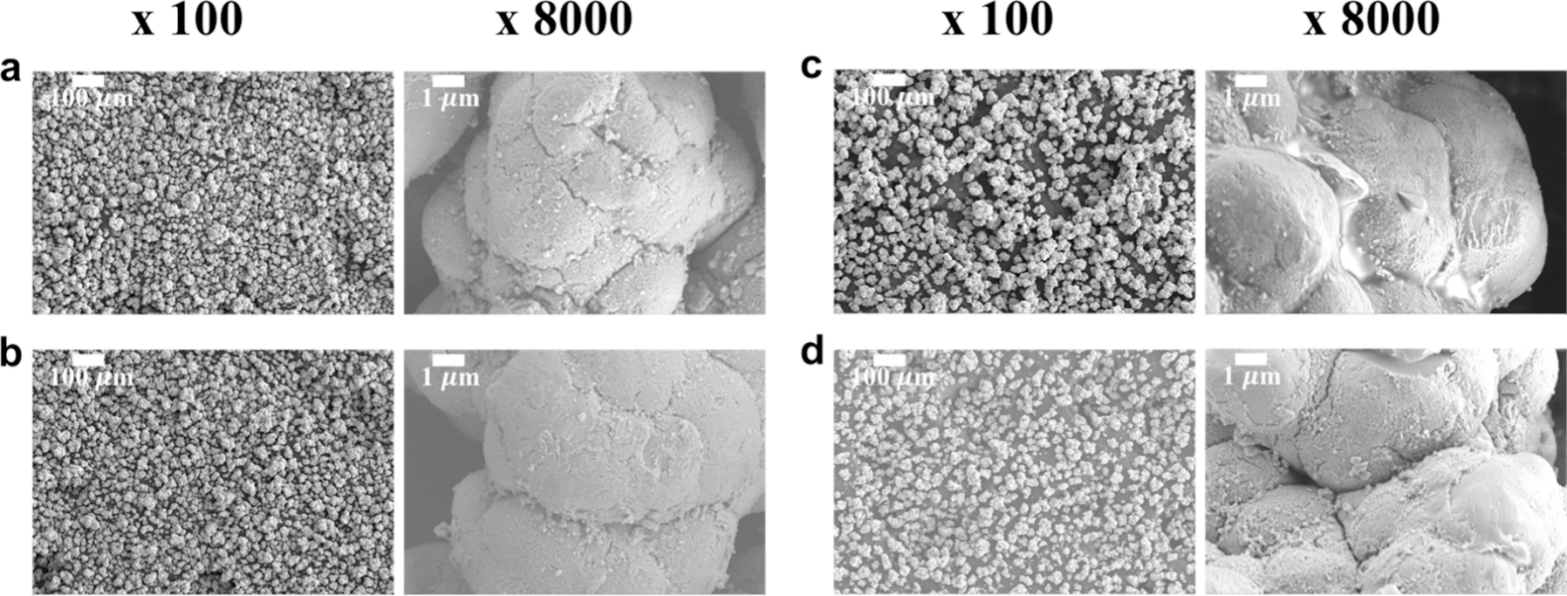
SEM images of uncoated ZrP (a), T (b), F12h (c), and TF12h (d) at 100× and 8000× magnification. “T” refers to ZrP coated with TEOS in liquid phase, while “F12h” represents ZrP coated with FOTS in vapor phase for 12 h. “TF12h” is ZrP coated with TEOS in liquid phase followed by FOTS in vapor phase for 12 h.

SEM images in Figure 3 reveal the surface morphologies of ZrP particles with different coatings. Uncoated ZrP in Figure 3a shows a porous surface texture, and its average particle size is ∼30 μm. Coated ZrP materials (T and TF12h) in Figure 3b,d have similar surface appearance to uncoated ZrP without considerable particle aggregation. The F12h without TEOS coating in Figure 3c shows some degree of aggregation, and the FOTS coating is visible on the ZrP surface, which is consistent with XPS results, suggesting FOTS alone gives rise to a much thicker coating layer. The invisibility of FOTS on TEOS-coated ZrP indicates that a thinner layer of FOTS was deposited on the TEOS coating than it was on bare ZrP. This is because TEOS coating provides a surface with higher density of OH groups that the FOTS molecules with three reactive hydrolyzable groups can be grafted with, instead of self-polymerization, which has been reported in other studies.^20^ Without the TEOS coating, the oligomeric FOTS, when attached to the ZrP surface, will yield a thick layer and be observed in SEM images.

### 3.2 NH_4_^+^ and Ca^2+^ Binding Study

**Figure 4:**
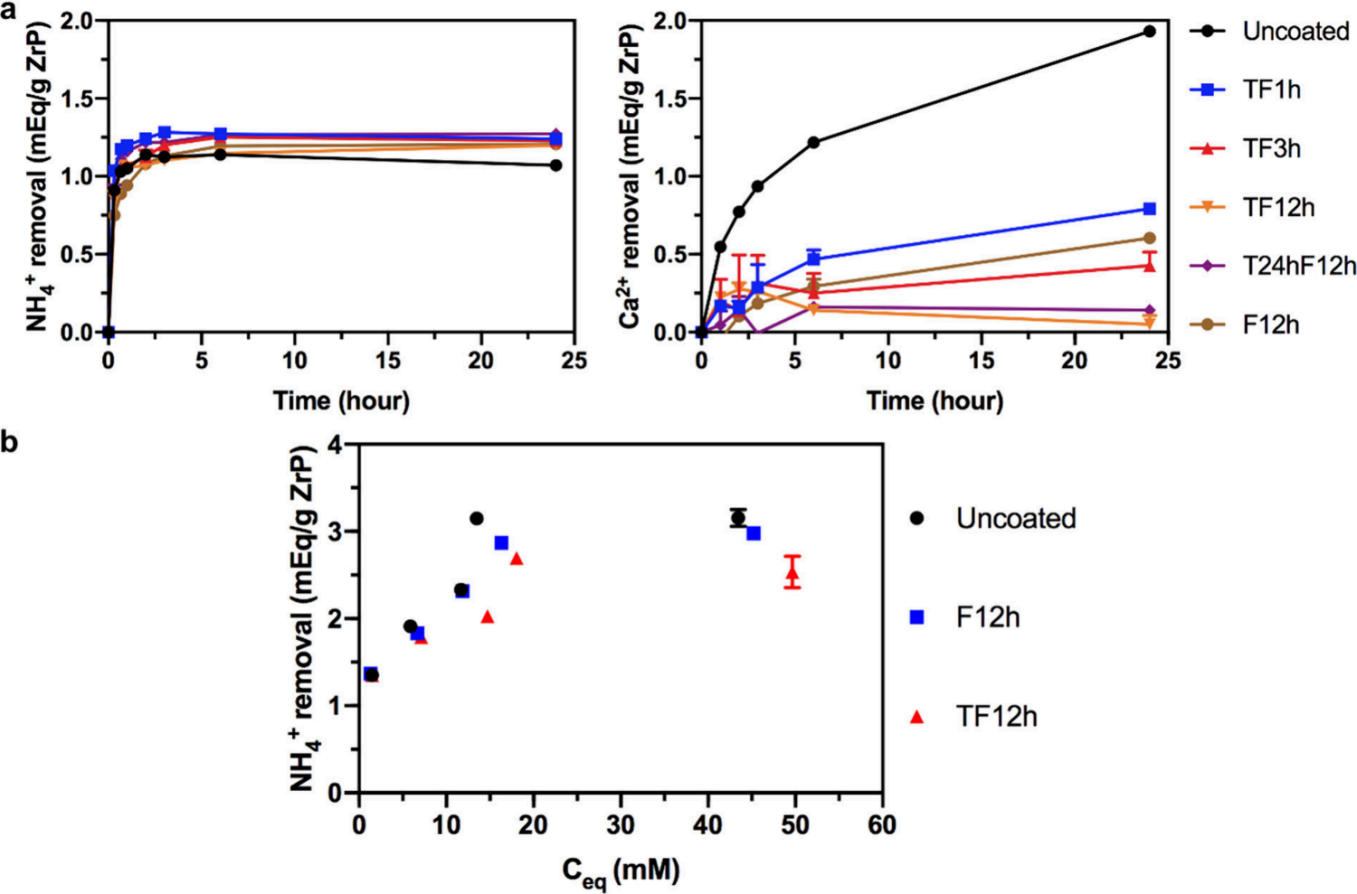
Competing ion binding study of uncoated and coated ZrP materials in a batch of water solution (15 mM NH_4_^+^, 15 mM Ca^2+^, and 20 mM HEPES) (a) and equilibrium binding study of uncoated ZrP, F12h, and TF12h in stock solutions with a single ion (15/25/35/45/75 mM NH_4_^+^) (b). Each data point was determined based on at least 3 sampling experiments.

Figure 4a provides the results of an *in vitro* competitive ion study of uncoated and coated ZrP in a fixed amount of water solution containing 15 mM NH_4_^+^ and Ca^2+^. Uncoated ZrP removes ∼1.14 mequiv of NH_4_^+^/g of ZrP from the solution within the first 2 h of the study, but by 24 h the removal decreases somewhat to ∼1.07 mequiv/g. In the meantime, Ca^2+^ removal by uncoated ZrP during 24 h is ∼1.93 mequiv of Ca^2+^/g of ZrP. The Ca^2+^ binding to the ZrP surface can replace adsorbed NH_4_^+^, which is released into the water solution, causing the decrease in NH_4_^+^ removal, since ZrP has a higher affinity for bivalent ions than monovalent ions.^5,12^ All TVD-coated ZrPs have similar NH_4_^+^ equilibrium binding to uncoated ZrP. At equilibrium, they can remove ∼1.2 mequiv NH_4_^+^/g, and there is no decline in NH_4_^+^ removal due to the coating’s blockage of transfer of Ca^2+^. However, TF12h (TEOS wet coating) and T24hF12h (TEOS TVD coating) are the only two materials that remove nearly zero Ca^2+^ from solution (0.05 and 0.14 meqiv/g, respectively), while TF1h, TF3h, and F12h all remove greater than 0.4 mequiv of Ca^2+^/g of ZrP. This demonstrates the importance of TVD duration of FOTS coating and presence of TEOS coating. Longer TVD duration yields more complete surface coverage of the FOTS coating (verified by higher F concentration in XPS analysis above); thus, water with Ca^2+^ is less likely to penetrate the coating and reach the ZrP surface, leading to negligible removal of Ca^2+^ from solution. A TEOS coating, according to previous studies,^5,12^ can provide abundant hydroxyl groups on the ZrP surface, which is the key for a complete FOTS layer in the next coating step. For materials without a TEOS coating, such as F12h, water with Ca^2+^ is allowed by more coating defects to penetrate the FOTS layer even though F12h is prepared with a longer TVD duration, leading to a higher degree of Ca^2+^ binding. The effectiveness of T24hF12h on blocking Ca^2+^ indicates that TEOS can also be coated on the ZrP surface in the vapor phase.

Although Ca^2+^ adsorption on ZrP has been significantly reduced by the TVD coating, a higher NH_4_^+^ removal was not achieved in our competitive binding study. Thus, the ammonium binding capacity of ZrP was tested in solutions with different concentrations of a single ion (NH_4_^+^). Figure 4b presents the removal of NH_4_^+^ on uncoated and coated ZrP versus the equilibrium NH_4_^+^ concentration. The results indicate that the NH_4_^+^ removal by ZrP materials is proportional to the NH_4_^+^ concentration in stock solution. The uncoated ZrP can remove ∼3.1 mequiv NH_4_^+^/g at an equilibrium concentration of ∼15 mM, while F12h and TF12h remove respectively ∼2.9 and ∼2.7 mequiv NH_4_^+^/g at equilibrium. The slightly lower ammonium binding by coated materials implies that some binding sites are likely blocked by the gas-permeable and hydrophobic membrane from adsorption of NH_4_^+^. In addition, the increase in sodium concentration within the ZrP capsule due to a restraining effect of the membrane could also lower NH_4_^+^ removal, while for uncoated ZrP the sodium releases into stock solutions. On the other hand, F12h and TF12h have the same equilibrium binding, suggesting the effect of particulate surface area on binding capacity is insignificant, because gaseous NH_3_ is highly diffusible.

**Figure 5:**
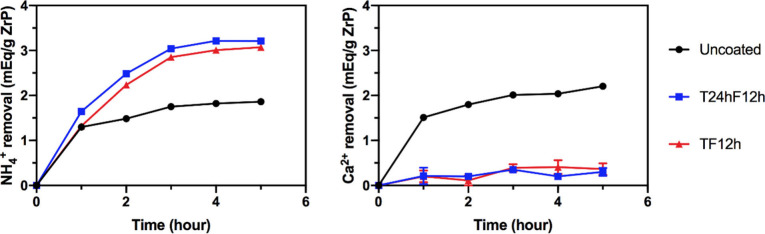
Competing ion binding study of uncoated and coated ZrP materials in a continuously replaced (every 20 min) water solution (14 mM NH_4_^+^, 12 mM Ca^2+^). “TF12h” refers to ZrP coated with TEOS in liquid phase followed by FOTS in vapor phase for 12 h, while “T24hF12h” was ZrP coated by TEOS in vapor phase for 24 h followed by FOTS in vapor phase for 12 h.

Based on static binding studies shown in Figure 4, coated ZrP materials, i.e., TF12h and T24hF12h, have nearly zero Ca^2+^ binding, and their NH_4_^+^ removal is dependent on NH_4_^+^ concentrations in solution. In the next step, a binding study has been conducted in a more dynamic way where a water solution exposed to ZrP materials is replaced every 20 min to keep NH_4_^+^ and Ca^2+^ concentration relatively constant. This could also be considered as a simulated small intestine physiological condition of ESKD patients.^5^Figure 5 presents the results of this binding study. Uncoated ZrP cumulatively removes ∼1.86 mequiv NH_4_^+^/g ZrP by 5 h; meanwhile TF12h and T24hF12h remove ∼3.1 and ∼3.2 mequiv NH_4_^+^/g ZrP, respectively. The improvement in the NH_4_^+^ binding capacity of coated materials is much more significant with this testing method, and the equilibrium NH_4_^+^ binding is consistent with that obtained from the equilibrium binding study in Figure 4b. On the other hand, these two coated ZrP materials remove only ∼0.3 mequiv Ca^2+^/g ZrP by 5 h, which is a huge improvement compared to ∼2.2 mequiv Ca^2+^/g ZrP removed by uncoated ZrP. Therefore, ZrP with a gas-permeable and hydrophobic coating formed in the vapor phase has been demonstrated with excellent NH_4_^+^ binding selectivity and capacity.

The maximum NH_4_^+^ binding capacity achieved in equilibrium and the dynamic binding study has not reached the maximum indicated in binary binding studies (∼6 mequiv/g) for ZrP yet.^36−38^ One reason is that our ZrP is partially Na-loaded, evidenced in the above XPS results where ∼5% sodium is present on the uncoated ZrP surface. ZrP total binding capacity could reach the maximum if ZrP is fully loaded with hydrogen because a lower pH within the ZrP capsule can facilitate the transfer of NH_3_ across the membrane. The NaOH titration curves in Figure S1 show a pH of ∼7.3 for a Na-loaded ZrP solution and a pH of ∼3.8 for a H-loaded ZrP solution. With H-loaded ZrP, gaseous NH_3_ can bind to the H^+^, and NH_4_^+^ forms within the ZrP capsule, where the pH increases but not as much as with Na-loaded ZrP, which has internal accumulation of Na^+^ and thus an increased pH.

### 3.3 Improved NH_4_^+^ Binding Capacity with H-Loaded ZrP

**Figure 6:**
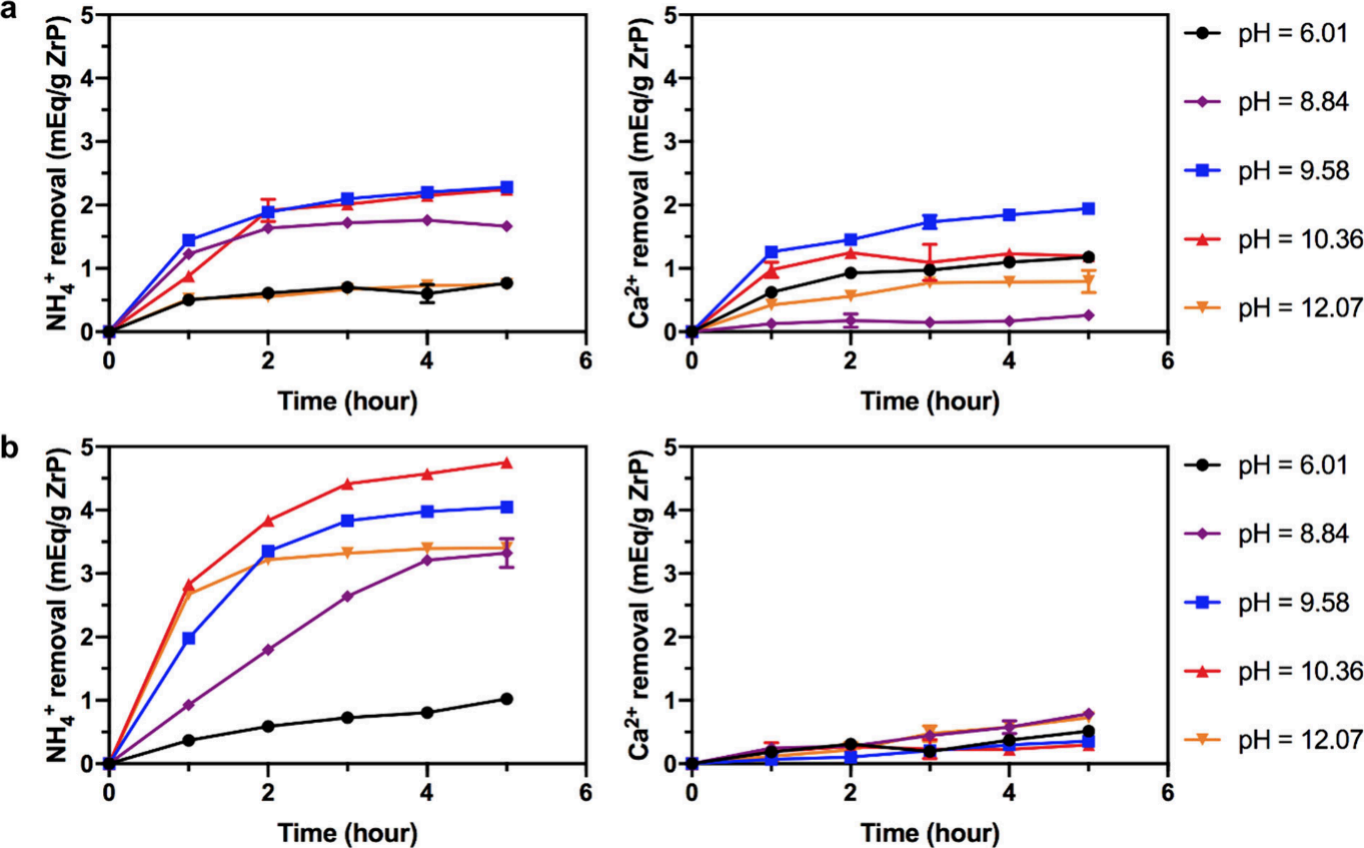
Competing ion binding study of uncoated ZrP (a) and T24hF12h (b) with hydrogen loading in a continuously replaced (every 20 min) water solution (14 mM NH_4_^+^, 12 mM Ca^2+^, 0/3/7.6/14/28 mM NaOH).

To find a sorbent with higher NH_4_^+^ binding capacity, we switched to study a H-loaded ZrP by applying the same coating condition (T24hF12h) and performing the binding study with continuously replacing the stock solution. Stock solutions with different concentrations of NaOH were prepared to investigate the effect of solution pH, which could influence the gradient of NH_3_ across the membrane and thus the NH_4_^+^ concentration within the capsules and the resulting binding capacity. As a result, uncoated ZrP and T24hF12h both have a limited NH_4_^+^ removal (<∼1 mequiv/g) by 5 h with a solution pH of ∼6 (without NaOH addition), shown in Figure 6a,b, indicating acidic conditions in solution inhibits the amounts of NH_3_ because NH_4_^+^ is in equilibrium with NH_3_ and H^+^ in any solution. With the addition of NaOH at concentrations of ∼3, ∼7.6, and ∼14 mM, uncoated ZrP can remove ∼2 mequiv NH_4_^+^/g by 5 h, and T24hF12h exhibits improved NH_4_^+^ binding capacities, which are above ∼3 mequiv/g. A NH_4_^+^ removal of 3.3 mequiv/g can be achieved at a pH of 8.84, which is close to the pH in the small bowel (pH ≈ 8) and dialysate from regeneration columns (pH = 8–9). In solution with pH values of 9.58 and 10.36 (∼7.6 and ∼14 mM NaOH), T24hF12h can remove ∼4 and 4.8 mequiv/g, respectively, by 5 h, showing the potential of H-loaded ZrP to achieve higher NH_4_^+^ binding compared to that (∼3.2 mequiv/g) from Na-loaded ZrP. Although these pH values are higher than realistic conditions, ZO-OH loaded with hydroxide can be added with sorbent materials to increase the gut pH to promote the NH_4_^+^ removal by coated ZrP.

The effect of solution pH on H-loaded ZrP ion exchange capacity has been reported previously.^36−44^ In general, the ion binding capacity of ZrP can be improved by increasing the solution pH level. At higher pH, hydrogen ions released from ZrP can be neutralized by OH^–^ in solution, while in acidic solution with lower pH, the buildup of released hydrogen will lower the ion exchange capacity because hydrogen ion can also compete for ion sorption.^5,12^ Here, the NaOH added in stock solution acts as a buffer to deplete H^+^ in solution and release it from ZrP, such that the equilibrium *NH*_4_^+^ ↔ *NH*_3_ + *H*^+^ will shift to the right to have abundant amount of NH_3_ to be removed by coated ZrPs. When the pH is raised to >12, the NH_4_^+^ binding capacity of T24hF12h during 5 h decreases to ∼3.4 mequiv/g because H^+^ within the ZrP capsule and TVD coatings may diffuse to the stock solution given the higher pH gradient. Thus, the amount of H^+^ remaining inside the ZrP capsule will become less for NH_3_ to adsorb. In other words, the higher pH within the ZrP capsule leads to a lower NH_4_^+^ removal. The Ca^2+^ removal of T24hF12h is only ∼0.35 mequiv/g at a pH of 10.36, which is significantly lower than the removal (>1.1 mequiv/g) of uncoated ZrP. Thus, the NH_4_^+^ binding capacity and selectivity have been effectively improved by using H-loaded ZrP with TVD coatings.

### 3.4 Coating Resistance to Acid Exposures

**Figure 7:**
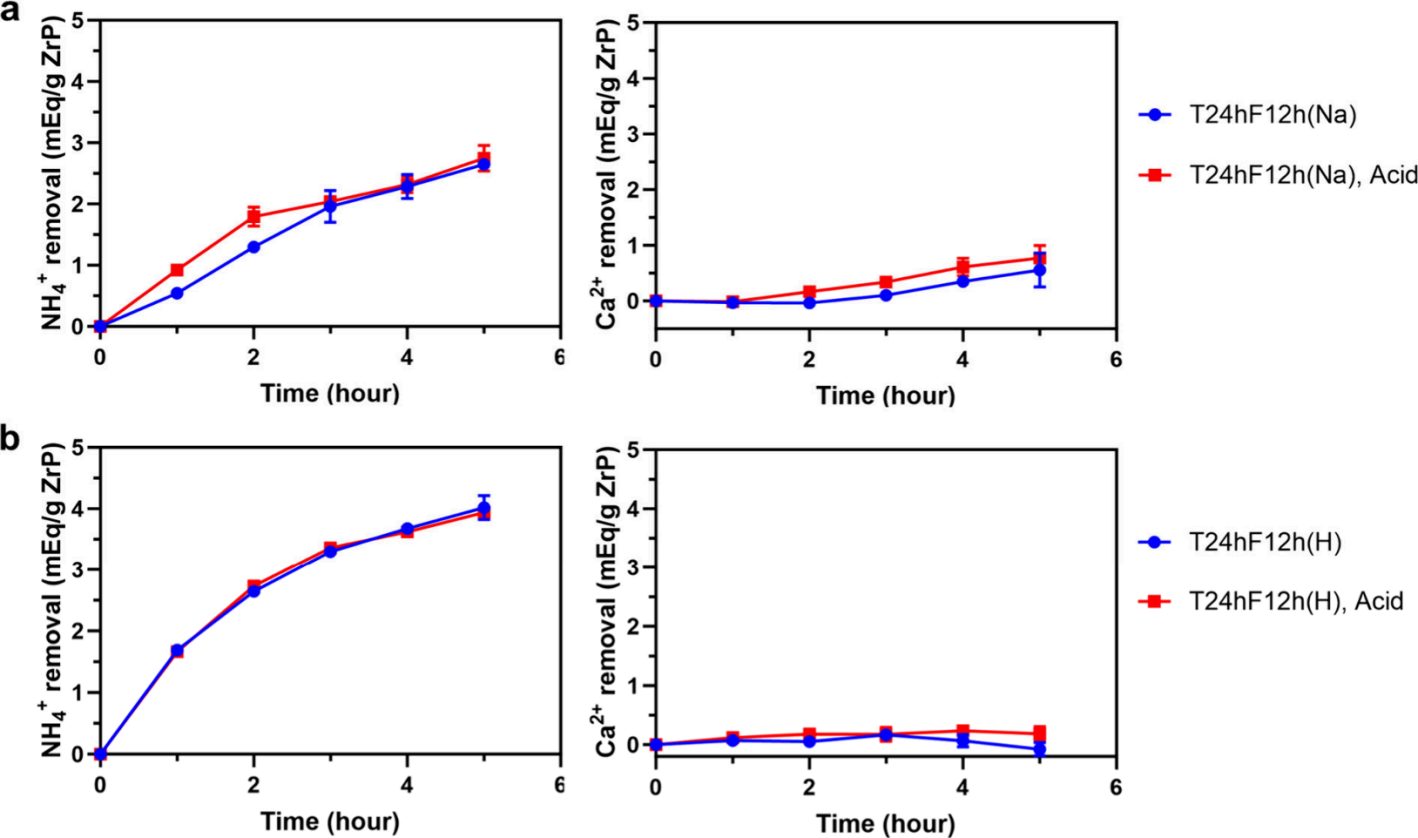
Competing ion binding study of Na-loaded (a) and H-loaded (b) ZrP with coating after acid exposure (solution pH is 5.9 for Na-loaded ZrP and 10.9 for H-loaded ZrP).

The resistance of ZrP as an oral sorbent to acidic solution is critical because it will pass through the stomach, which is a low-pH environment (pH = 2–3), before entering the small intestine. Figure S4 shows that there is no significant change for coated ZrP materials in SEM images, XPS atomic composition, and WCA after acid treatment. Figure 7a,b shows the results of the dynamic binding study on coated materials (a: Na-loaded T24hF12h in a solution of 14 mM NH_4_^+^ and 12 mM Ca^2+^; b: H-loaded T24hF12h in a solution of 14 mM NH_4_^+^, 12 mM Ca^2+^, and 14 mM NaOH) before and after acid treatment (pH = 2). The Na-loaded and H-loaded T24hF12h can remove respectively ∼2.8 and ∼4 mequiv NH_4_^+^/g in 5 h, which are not adversely impacted by the acid treatment. The lower NH_4_^+^ removal (∼4 mequiv/g) of H-loaded ZrP than that reported in the previous section (∼4.7 mequiv/g) is because of the slight difference in pH of stock solutions in the two binding studies (10.9 vs 10.36). As explained above, a pH too high in the stock solution could also decrease the NH_4_^+^ removal. On the other hand, the ability of acid-treated materials to remove Ca^2+^ does not change significantly (only ∼0.2 mequiv Ca^2+^/g increase compared to results before acid treatment), which means that the NH_4_^+^ removal selectivity does not decline and that TVD coatings on the two ZrP materials remain intact in an acid environment. FOTS has been prominent with its chemical stability and inertness,^45^ and it has three reactive groups and thus a high degree of cross-linking, which is more stable in acid exposure. The outstanding acid resistance of T24hF12h materials suggests that ZrP with the gas-permeable and hydrophobic membrane formed in the vapor phase is promising as an oral sorbent for ESKD patients.

## 4 Conclusion

This study has successfully developed a solventless coating protocol that effectively coats ZrP with TEOS and FOTS via vapor phase deposition, which significantly reduces the preparation length compared to the wet chemistry approach and eliminates the usage of solvents such as acetone. The TVD duration was optimized based on the coated materials’ ability to selectively remove NH_4_^+^ in a static binding study. The dynamic *in vitro* binding study shows a maximum NH_4_^+^ binding capacity of ∼3.2 mequiv/g for Na-loaded ZrP, which can be further improved by replacing Na-loaded ZrP with H-loaded ZrP for TVD coatings (∼4.7 mequiv/g), and both coated materials barely remove Ca^2+^ in the meantime. FOTS-coated ZrP materials by TVD also exhibit excellent resistance in acid treatment. H-loaded ZrP is thus a suitable material for TEOS and FOTS coating by TVD, and the coated ZrP could serve as an oral sorbent to diminish BUN levels in patients with ESKD. It may also serve as a sorbent for NH_4_^+^ generated by urease in columns used for regeneration of dialysate. Moving forward, *in vitro* tests are needed to determine whether the coatings are solubilized in a water suspension or fouled or degraded by some biological components of the gut such as acid, protein, and bile salts. These studies will indicate whether it is reasonable to proceed to animal trials of this potentially valuable oral sorbent.

## Supporting Information Available

NaOH titration curve for Na-loaded and H-loaded ZrP; atomic surface composition, SEM images, and water contact angle of H-loaded and Na-loaded ZrP materials at different coating conditions before and after acid exposure (PDF)
