# Supplementary material for: Thermal Vapor Deposition of a Hydrophobic and Gas-Permeable Membrane on Zirconium Phosphate Cation Exchanger: An Oral Sorbent for the Urea Removal of Kidney Failure
Source: Langmuir. 2024 Jul 23;40(31):16502–10. doi: 10.1021/acs.langmuir.4c01877 (PMC11308767; doi:10.1021/acs.langmuir.4c01877)
Supplement: Supplementary file 1 — la4c01877_si_001.pdf [file la4c01877_si_001.pdf]

Thermal Vapor Deposition of a Hydrophobic and Gas-Permeable Membrane on Zirconium Phosphate Cation Exchanger: An Oral Sorbent for Urea Removal of Kidney Failure.

Yihan Song,[1] Sang-Ho Ye,[2] Stephen R. Ash,[3*] and Lei Li[1*]

1. Department of Chemical and Petroleum Engineering, University of Pittsburgh, Pittsburgh, Pennsylvania 15260, United States

2. McGowan Institute for Regenerative Medicine, Pittsburgh, Pennsylvania 15219, United States; Department of Surgery, University of Pittsburgh, Pittsburgh, Pennsylvania 15260, United States

3.Nephrology Department, Indiana University Health Arnett Hospital, Lafayette, Indiana 47905, United States; CEO, HemoCleanse Technologies, LLC, Lafayette, Indiana 47904, United States

*: Corresponding Authors:

Stephen R. Ash (Email: sash@hemocleanse.com); Lei Li (Email: lel55@pitt.edu)

Experimental Procedure:

NaOH titration: 0.1 M NaOH solution is prepared, and ~50 mg Na-loaded and H-loaded ZrP were suspended in 5 mL DI water respectively. At each titration point, 0.2 mL NaOH solution was added into ZrP suspension, and pH was measured after equilibration for ~30 min using a pH meter (Oakton, pH 6 Acorn series).

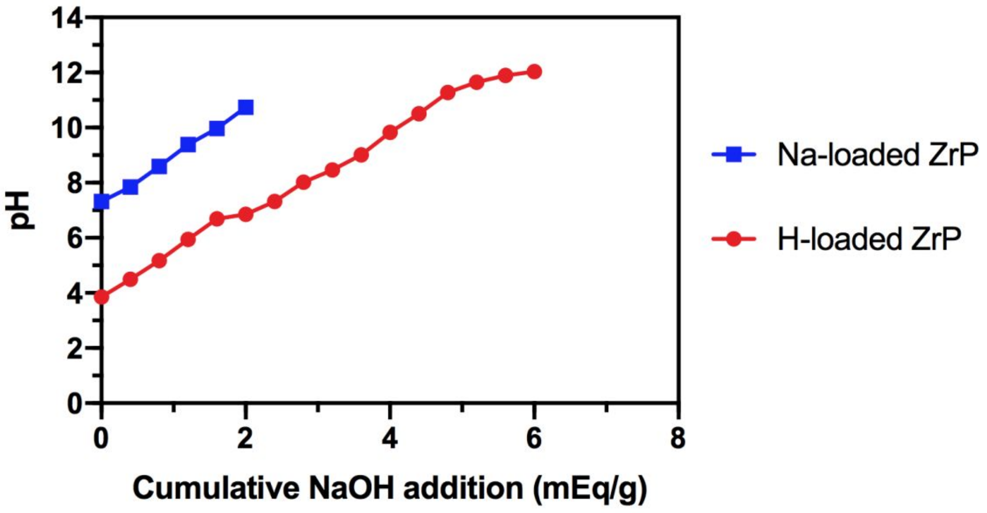

Figure S1. NaOH titration curve for Na-loaded and H-loaded ZrP.

The NaOH titration curves in Figure S1 show a pH of ~7.3 for Na-loaded ZrP solution and a pH of ~3.8 for H-loaded ZrP solution, which indicates H-loaded ZrP has potential for higher NH_4^+ binding capacity.

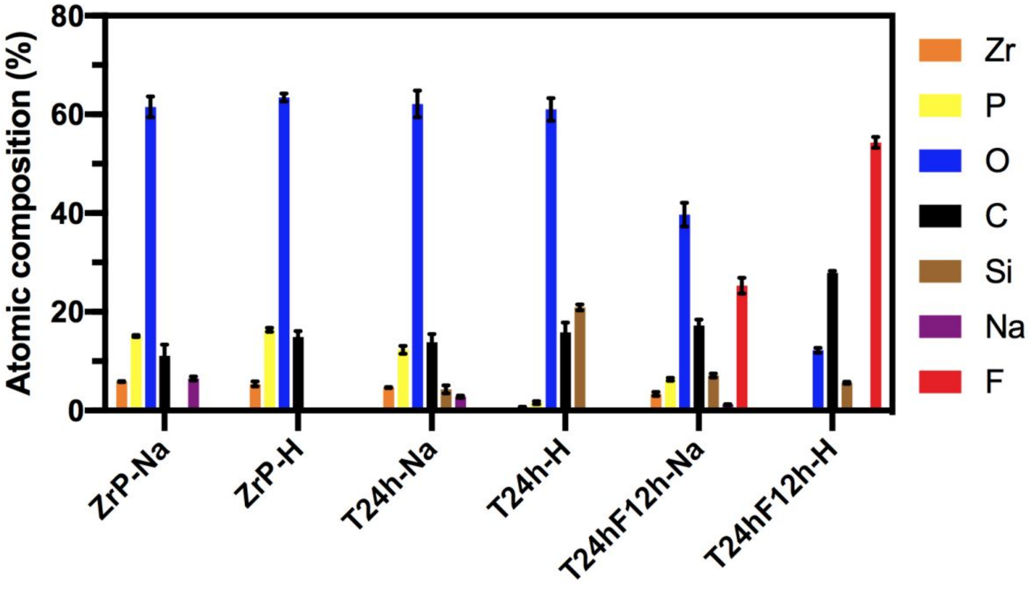

Figure S2. Atomic surface composition of ZrP materials at different coating conditions.

The absence of Na on ZrP-H indicates a fully hydrogen loaded ZrP material. After TEOS coating, more Si was detected on H-loaded ZrP than Na-loaded ZrP, suggesting a thicker TEOS layer formation given same TVD duration (24h). This is possibly because H-loaded ZrP is more hydrated than Na-loaded ZrP, and more water promotes a higher degree of hydrolysis of TEOS reactive groups. After FOTS coating, the surface of H-loaded ZrP has more F than that of Na-loaded ZrP (~54% vs. ~25%), suggesting a thicker FOTS coating formed. The absence of Zr and P indicates the TVD coatings on H-loaded ZrP is at least 10 nm.

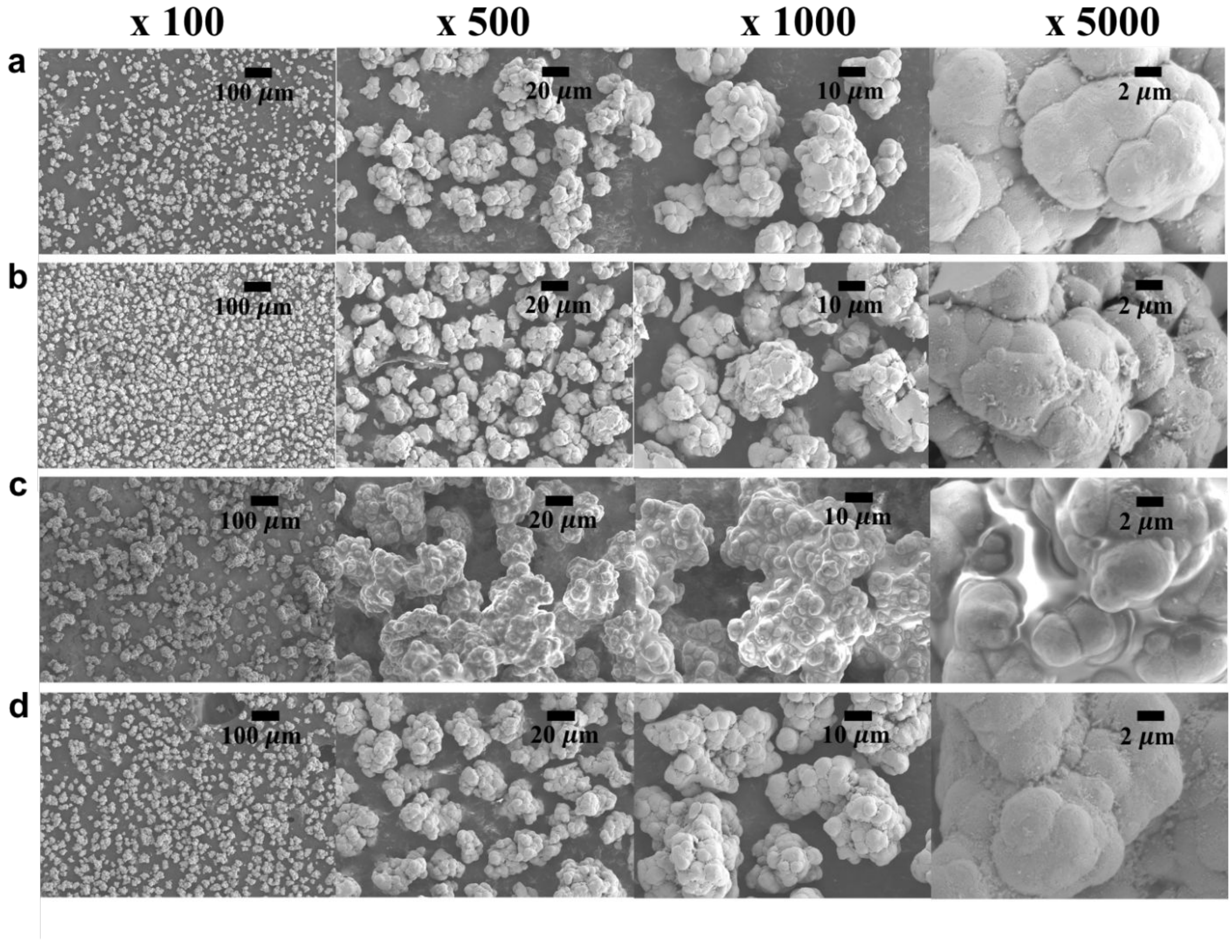

Figure S3. SEM images of uncoated H-loaded ZrP (a), T24h H-loaded ZrP (b), T24hF12h H-loaded ZrP (c) and T24hF12h Na-loaded ZrP (d) at 100x, 500x, 1000x and 5000x magnification. "T" or "F" was coated by TVD.

The SEM images of H-loaded ZrP in Figure S3a show an average particle size ~30 μm, which is not significantly different from Na-loaded ZrP. After TEOS coating, some textured features are found on H-loaded ZrP surface, which is possibly ascribed to oligomeric TEOS in the thicker coating formed mentioned above. After FOTS coating, H-loaded ZrP exhibits significant particulate aggregation along with self-polymerization of FOTS precursors, in contrast to Na-loaded ZrP on which aggregation and FOTS are not detectable. This is consistent with XPS results where F concentration is much higher on H-loaded ZrP than Na-loaded ZrP, indicating a higher FOTS coating thickness on H-loaded ZrP.

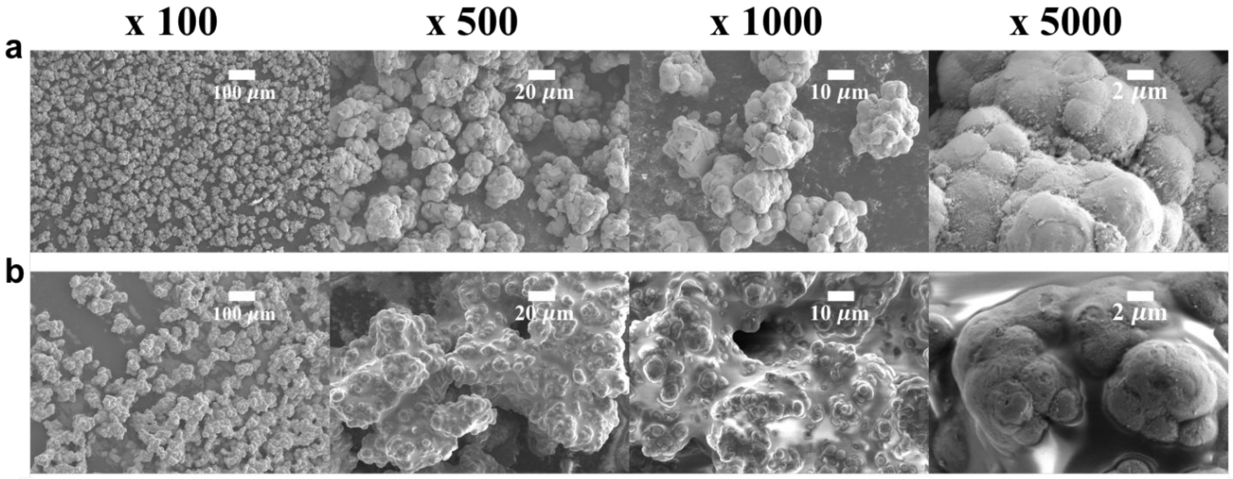

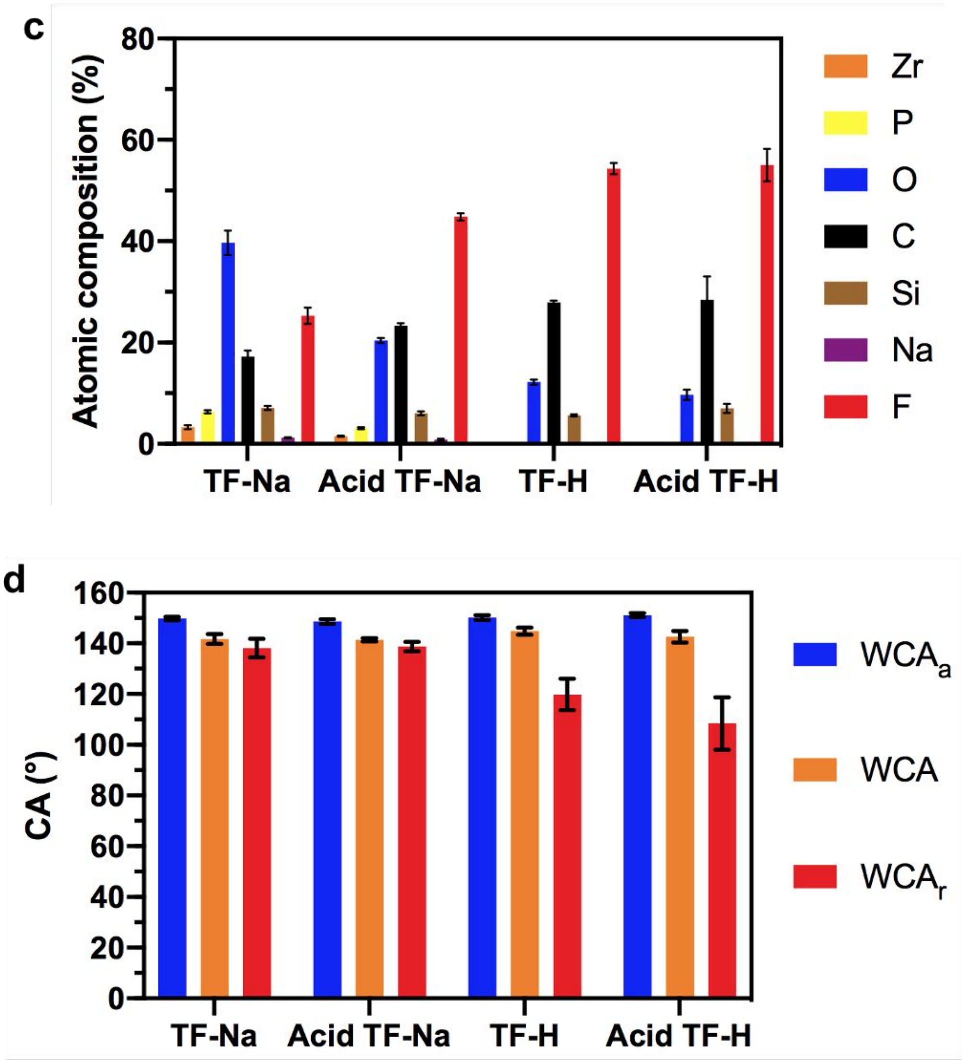

Figure S4. SEM images of Na-loaded (a) and H-loaded (b) ZrP with coatings after acid exposure, and atomic surface composition (c) and water contact angle (d) of ZrP materials before and after acid exposure.

The SEM images in Figure S4a-b present coated ZrP materials after acid treatment. No significant change is found compared to before acid treatment, which means our TVD coatings remain intact within acid exposure. As shown in Figure S4c, H-loaded ZrP with TVD coatings has unchanged surface composition before and after acid treatment, while Na-loaded ZrP shows a decreased oxygen concentration (from ~40% to ~20%) and an increased F concentration (from ~25% to

~44%). This can be explained by the fact that some TVD reaction byproducts on Na-loaded ZrP, e.g., H_2O and C_2H_5OH, are removed by acid treatment, but such change on a much thicker coating of H-loaded ZrP is not substantial. The unchanged performance of acid treated ZrP in binding study shown in Figure 7, and the water contact angle data in Figure S4d, indicate our TVD coatings have outstanding resistance to acid treatment.
